# Ferroptosis-Related lncRNA to Predict the Clinical Outcomes and Molecular Characteristics of Kidney Renal Papillary Cell Carcinoma

**DOI:** 10.3390/cimb46030123

**Published:** 2024-02-29

**Authors:** Yubo Gong, Chenchen Zhang, Hao Li, Xiaojie Yu, Yuejia Li, Zhiguo Liu, Ruyi He

**Affiliations:** 1 School of Life Science and Technology, Wuhan Polytechnic University, Wuhan 430023, China; yubogonglst@163.com (Y.G.); zcc2023056@163.com (C.Z.); 18871213061@163.com (H.L.); y965088253@126.com (X.Y.); 15527687109@163.com (Y.L.); 2 State Key Laboratory of Biocatalysis and Enzyme Engineering, School of Life Sciences, Hubei University, Wuhan 430062, China

**Keywords:** ferroptosis, lncRNA, KIRP, clinical outcome, risk model

## Abstract

Kidney renal papillary cell carcinoma (KIRP) is a highly heterogeneous type of kidney cancer, resulting in limited effective prognostic targets for KIRP patients. Long non-coding RNAs (lncRNAs) have emerged as crucial regulators in the regulation of ferroptosis and iron metabolism, making them potential targets for the treatment and prognosis of KIRP. In this study, we constructed a ferroptosis-related lncRNA risk score model (FRM) based on the TCGA-KIRP dataset, which represents a novel subtype of KIRP not previously reported. The model demonstrated promising diagnostic accuracy and holds potential for clinical translation. We observed significant differences in metabolic activities, immune microenvironment, mutation landscape, ferroptosis sensitivity, and drug sensitivity between different risk groups. The high-risk groups exhibit significantly higher fractions of cancer-associated fibroblasts (CAFs), hematopoietic stem cells (HSC), and pericytes. Drugs (IC50) analysis provided a range of medication options based on different FRM typing. Additionally, we employed single-cell transcriptomics to further analyze the impact of immune invasion on the occurrence and development of KIRP. Overall, we have developed an accurate prognostic model based on the expression patterns of ferroptosis-related lncRNAs for KIRP. This model has the potential to contribute to the evaluation of patient prognosis, molecular characteristics, and treatment modalities, and can be further translated into clinical applications.

## 1. Introduction

Kidney renal papillary cell carcinoma (KIRP) is the most heterogeneous form of renal cell carcinoma, with significant variations in survival rates among patients. There is currently no effective treatment for advanced KIRP [1]. In clinical studies, KIRP patients have demonstrated the second-highest morbidity rate among RCC cases, along with a poor prognosis [2,3]. Presently, radical or partial nephrectomy continues to be the standard treatment for KIRP patients; however, the recurrence rate remains high at nearly 40%. Therapeutic targets for KIRP have been identified by researchers, including cabozantinib, which specifically targets type 1 KIRP and not the more aggressive type 2 KIRP [4]. KIRP patients with poor survival rates exhibit molecular characteristics such as metabolic disorders, immune disorders, and CpG island methylation phenotype (CIMP). Currently, there is still a lack of reliable biomarkers for the effective clinical treatment of KIRP [5]. The majority of anti-tumor drugs used in clinical practice achieve therapeutic goals by inducing cell death in cancer cells. Therefore, it is essential to utilize alternative forms of cell death to combat drug resistance in tumor cells for early tumor detection and treatment.

Ferroptosis is an iron-dependent regulatory cell death mechanism that is triggered by oxidative stress. It can be inhibited by iron chelators and lipophilic antioxidants and is associated with the development and treatment response of various types of tumors [6]. It differs from apoptosis, necrosis, autophagy, and other forms of cell death in terms of morphology, biochemistry, and genetics. There are reports that ferroptosis can exacerbate kidney injury, heart failure, bone marrow injury, brain injury, spinal cord injury, and intestinal ischemia/reperfusion injury [7,8,9,10,11]. Several studies have confirmed the key role of ferroptosis in tumor development and treatment [12,13,14]. Ferroptosis-related genes (FRGs) such as *p53* [15], Fanconi anemia complementation group D2 (*FANCD2*) [16], and Dipeptidyl peptidase 4 (*DPP4)* [17], play an important role in tumorigenesis and development. Additionally, many tumors are sensitive to ferroptosis, including ovarian cancer, adrenocortical carcinoma, lung adenocarcinoma, and hepatocellular carcinoma cells [18,19,20,21]. According to NCI-60 (a collection of 60 cancer cell lines used for screening new anticancer drugs under the regulations of the National Cancer Institute in the United States), research indicates that renal cancer cells and leukemia cells are more susceptible to clearance compared to cancer cells from the lungs, colon, central nervous system, melanocytes, ovaries, and breasts. This suggests that targeted therapy focusing on ferroptosis may have more benefits in treating renal cancer [21]. Given the severe shortage of effective drugs for the clinical treatment of KIRP, there is an urgent need for new drug targets. Therefore, ferroptosis is considered a promising approach for treating KIRP as it is a targeted and effective process for killing tumors [22].

Numerous studies have found that lncRNA can regulate the biological behavior of KIRP [23,24,25,26]. Studies have shown that lncRNA can affect ferroptosis by directly interacting with proteins or inhibiting translation processes. This indicates that lncRNA plays multiple roles in regulating ferroptosis [27]. Metabolic transformation is a hallmark of cancer and a critical target for cancer treatment. The metabolism and behavior of tumors are regulated by intracellular factors and the availability of metabolites in the tumor microenvironment. Therefore, identifying lncRNAs related to ferroptosis is of great significance for deciphering the potential mechanisms of the KIRP tumor microenvironment and in terms of the search for new therapeutic targets [28]. 

In this study, we constructed a 9-lncRNAs FRM, which showed promising diagnostic accuracy for potential clinical translation. The area under the curve was 0.91 for the 3-year survival rate. In addition, we found that *ACSF2* is a potential prognostic factor for renal cancer. The metabolic activities, immune microenvironment, mutation landscape, ferroptosis sensitivity, and drug sensitivity were significantly distinct between patients with different risk scores. We also used single-cell transcriptomic analysis to examine the FRM at the cellular level. The results revealed that upregulated genes in the high-risk group are largely found in proliferating tumor macrophages, while downregulated genes are found in renal tubular cells. These findings have significant implications for assessing patient prognosis, understanding molecular characteristics, determining treatment approaches, and potentially translating them into clinical applications.

## 2. Results

### 2.1. Identify FRLs Using Bioinformatic Approaches

**Figure 1:**
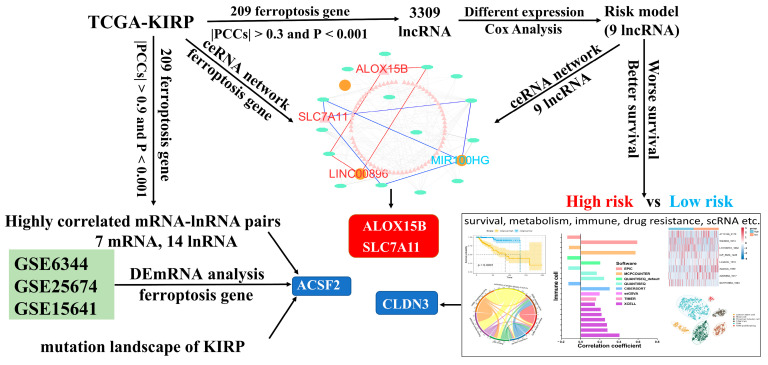
The flowchart of this study.

The graphic flowchart displayed the main design of the present study in Figure 1. The transcriptomic expression levels of 241 FRGs and 3680 lncRNAs were extracted from 321 resected KIRP samples in the TCGA database. These expression levels were then integrated into a co-expression matrix. According to the screening criteria (r > 0.3 and *p* < 0.001), 3309 FRLs were identified. A total of 349 differentially expressed FRLs (DEFRLs) were identified between cancer and normal tissues, with 234 being downregulated and 115 being upregulated. Additionally, 208 FRGs associated with these DEFRLs were found to be involved in metabolic reprogramming (Figure S1). Notably, some lncRNAs were highly correlated with the expression of seven core ferroptosis regulators: *ACSF2*, *ANGPTL7*, *DUOX2*, *IFNG*, *PCK2*, *SOCS1*, and *TP63* (Figure S2A). GEO RNAseq data analysis indicated that the expression of FRG *ACSF2*, *CA9*, *LOX*, *DDIT4*, and *CAV1* was differentially regulated in three data groups. Among these genes, only *ACSF2* was downregulated and highly correlated with the lncRNA LINC01020 (Figure S2B). Thus, *ACSF2* might play a crucial role in regulating ferroptosis in renal cell carcinoma. However, no studies have investigated the role of *ACSF2* in kidney cancer. [PubMed keyword research: (((ACSF2) AND (kidney)) OR (renal carcinoma)) OR (renal cell carcinoma)]. *ACSF2* (acyl-CoA synthetase family member 2) is a well-known molecule that drives ferroptosis and is involved in the regulation of mitochondrial fatty acid metabolism [29]. Notably, *ACSF2* was found to have a positive association with *LINC01020* expression in three types of kidney cancer (KIRP r = 0.90, *p* < 0.0001, KICH r = 0.81, *p* < 0.0001, KIRC r = 0.69, *p* < 0.0001) (Figure S2C). Next, we analyzed the differential expression of KIRP in pan-cancer and found that the expression of *ACSF2* was low in all three types of RCC. Until now, no studies have reported the role of *ACSF2* or *LINC01020*, or their interaction, in ferroptosis or kidney tumor prognosis. However, *ACSF2* has shown significant differential expression in most tumors (Figure S2D).

### 2.2. Construction of FRMto Predict the OS of Patients with KIRP

**Figure 2:**
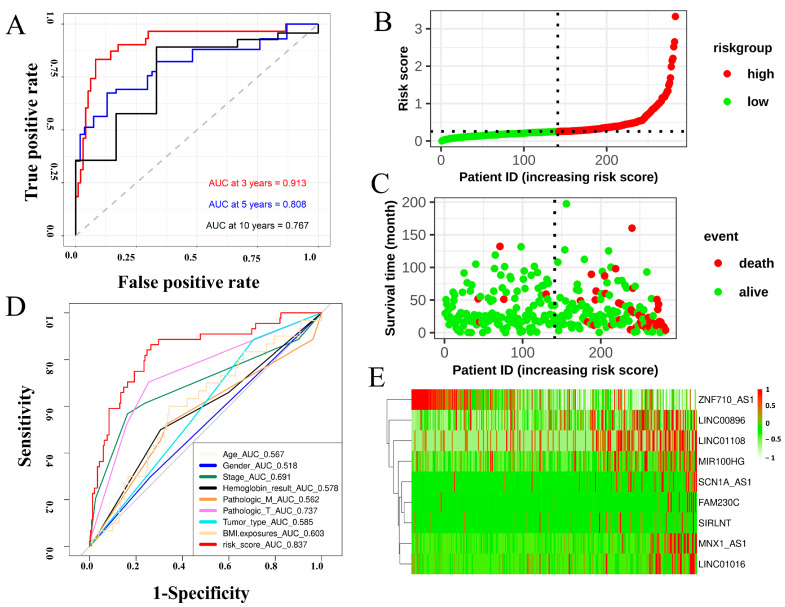
Construction of a prognostic model to predict the survival expectancy of patients with KIRP. (**A**) ROC curve depicting the 3-, 5-, and 10-year survival predictions of the FRM model. (**B**) Risk score of each individual. (**C**) Survival status and survival time of each individual. The color of each plot represents the survival status of each patient. (**D**) ROC indicated that the predictive accuracy of FRM was superior to other clinical parameters. Multivariate Cox regression analysis revealed that the FRM risk score was an independent risk factor for OS in patients with KIRP. (**E**) Heatmap showing differentially expressed predictive genes included in the risk model.

The transcriptomic expression matrix of 349 differentially expressed FRLs was selected with abs(logFC) > 2 and *p* value < 0.01 for the subsequent analysis. Univariate Cox regression was performed to screen for lncRNAs associated with overall survival (OS). Lasso regression was performed to calculate the coefficient of each lncRNA in the FRM. A total of nine lncRNAs were used in the FRM. The risk score was calculated using the following formula: Risk score = 0.0134976 × *SCN1A_AS1* + 0.0088863 × *MNX1_AS1* + 0.0584022 × *LINC01016* + 0.0110878 × *FAM230C* − 0.0012206 × *ZNF710_AS1* + 0.0013065 × *MIR100HG* + 0.0644311 × *SIRLNT* + 0.3185535 × *LINC01108* + 0.0136859 × *LINC00896*. The screening procedures described in the Methods section were followed to select these lncRNAs. The c-index of the model was 0.8724352. Based on the linear FRM risk scores, ROC curves were plotted to assess the predictive accuracy for overall patient survival (OS). Multivariate Cox regression analyses revealed that the risk model was an independent factor with prognostic value for predicting the OS of patients with KIRP. Furthermore, the later stage of cancer was associated with a higher number of high-risk patients (Figure S3A,B). The optimal cut-off value was defined as the risk score that reflected the largest area under the curve (AUC) value (Figure 2A). Patients were divided into two groups based on the optimal cut-off value of FRM risk for diagnostic accuracy (Figure 2B). More death events were observed in the high-risk group, suggesting that increased FRM risks were indicative of an unfavorable prognosis in patients with KIRP (Figure 2C). Kaplan–Meier curves of either the entire sample or randomly selected samples all showed a significant difference in OS between high and low FRM risks (*p* < 0.001, Figure S3C,D). Age, gender, AJCC stage, hemoglobin, tumor type, and BMI were comparable among the FRM risk groups (Figure 2D). Notably, the predictive capacity of these traditional clinical parameters was significantly weaker than that of the FRM risk score and risk group (Figure S3E). LncRNAs involved in FRM were all highly expressed, except for ZNF710-AS1 in the high-risk group (Figure 2E and Figure S4). The expression of *MIR100HG*, *MNX1_AS1*, and *LINC01016* increased in the later tumor stages (Stage III and Stage IV) (Figure S3F).

### 2.3. Differentially Expressed Genes Revealed Cell Metabolic Alterations Associated with FRM Risk in KIRP

**Figure 3:**
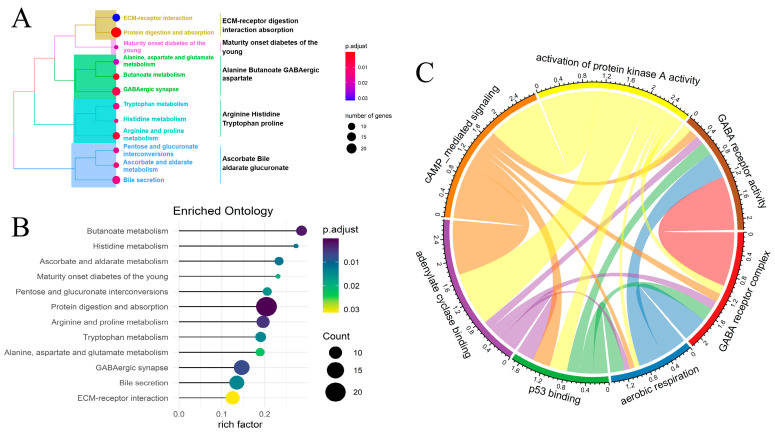
Comparison of the differentially expressed genes and pathways between the high- and low-risk groups in FRM. (**A**) KEGG analysis revealed the pathways primarily involved with the differentially expressed genes. (**B**) GSEA analysis of differentially expressed genes. (**C**) The correlation between differentially activated metabolic pathways. The size of each circle represents the *p* value for the differential expression analysis.

A total of 842 differentially expressed genes were identified between the FRM high- and low-risk groups. The criteria for selection were abs(logFC) > mean(abs(logFC)) + 2 × sd(abs(logFC)) and *p* value < 0.01 (Figure S5A,B). Gene ontology (GO) analysis revealed that most of the genes were enriched in extracellular activities, such as collagen-containing extracellular matrix, extracellular matrix organization, and anion transmembrane transporter activity (Figures S5C–E and S6). These activities influence cell behaviors such as proliferation, adhesion, and migration, and regulate cell differentiation and death [30]. KEGG and GSEA analysis demonstrated that these genes were involved in cell invasion and cell signaling. The pathways in which they were classified are as follows: ECM-receptor digestion interaction absorption, Alanine Butanoate GABAergic aspartate, Arginine Histidine Tryptophan proline, and Ascorbate Bile aldarate glucuronate (Figure 3A,B). The abnormal membrane transport mechanism influences the cellular microenvironment, leading to the occurrence and development of cancer. It can also affect the therapeutic effect, including cancer-related fatigue [31].

Next, we conducted a more in-depth analysis of the cell signaling pathway linked to FRM risk in KIRP samples. A total of 43 differentially activated metabolic pathways were identified (*p* < 0.05). Most of these pathways were found to be downregulated in the FRM high-risk group, while only four pathways were upregulated. These upregulated pathways include lipid droplet, neurofilament, GABA receptor activity, and the GABA receptor complex. On the other hand, pathways such as cyclic-nucleotide-mediated signaling, positive regulation of ATPase activity, and adenylate cyclase activity were found to be downregulated (Figure S7). We also analyzed the correlation between these pathways to reveal the mutual interactions in the cell signaling transport network (Figure 3C). We found that cell communication, particularly GABA receptor activity, the GABA receptor complex, aerobic respiration, p53 binding, adenylate cyclase binding, cAMP-mediated signaling, and activation of protein kinase A activity, play crucial roles in these two groups (the high/low-risk and tumor-normal groups), with distinct regulatory patterns. 

### 2.4. Differences in the Immune Microenvironment and Immune-Related Signatures between the High and Low-Risk Groups of FRM

**Figure 4:**
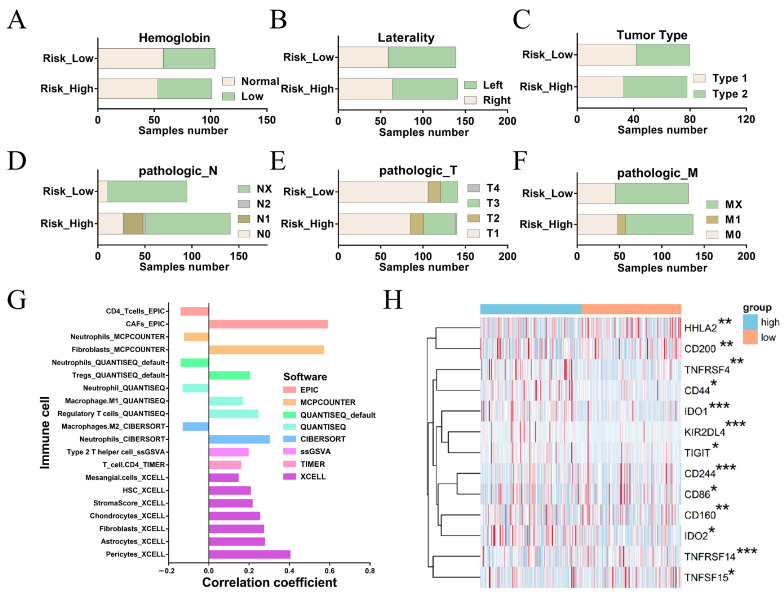
Differences in the immune microenvironment and immune-related characteristics between the high- and low-risk groups for FRM. (**A**–**F**) Composition of traditional KIRP molecular subtypes in FRM high-risk and low-risk groups. (**G**) Correlation between FRM risk and immune cell infiltration. (**H**) Association between FRM risk score and immune checkpoint gene expression levels. The significantly differentially expressed genes between high and low risk groups were shown. * *p* < 0.05, ** *p* < 0.01, *** *p* < 0.001. Blue indicates high-risk group and Orange indicates low-risk group.

We investigated the differences in molecular characteristics between the high- and low-risk groups of FRM. We compared the composition of traditional KIRP molecular subtypes in the two groups. The results showed that the sample number of each KIRP subtype was consistent with FRM-based clustering (*p* > 0.05, Figure 4A–F). This indicates that FRM produced an independent classification.

We next investigated the correlation between FRM risk and immune cell infiltration (Figure 4G). We found that FRM risk was positively associated with the components that could influence immune cell infiltration, including CAFs, fibroblasts, HSCs, and chondrocytes [32]. This suggests that increased FRM risk is linked to enhanced tumor immunosuppression.

The immune checkpoint gene (ICG) plays a key role in preventing self-reactivity and represents a promising avenue for the development of new targets in cancer therapy. We further examined the relationship between the FRM risk score and the expression level of immune checkpoint genes (Figure 4H). It is worth noting that IDO1, IDO2, CD44, and CD200 are up-regulated in the FRM high-risk group, all of which are related to the tumor microenvironment [33,34,35,36]. By breaking down tryptophan into N-formyl-kynurenine, IDO deprives T cells of the essential amino acid tryptophan, preventing them from mounting an effective immune response [37]. In addition, IDO produces soluble factors (kynurenine and downstream metabolites) that bind and activate the aryl hydrocarbon receptor (AhR, which can promote Treg cell differentiation) [38,39], and can also bias dendritic cells (DCs) and macrophages toward an immunosuppressive phenotype [40]. Thus, IDO inhibitors, such as GDC-0919 and Epacadostat [41], may be potential treatment options for high-risk patients. There is also an increase in CD44 and CD200 in the high-risk group. In contrast, HHLA2, a newly identified member of the B7 immune checkpoint family, was upregulated in the low-risk group of FRM (Figure 4H), suggesting that HHLA2 could be a potential therapeutic immune target in the low-risk KIRP group.

### 2.5. Comparison of the Differential Expression of Ferroptosis Regulators in KIRPs with Different FRM Risk Scores

**Figure 5:**
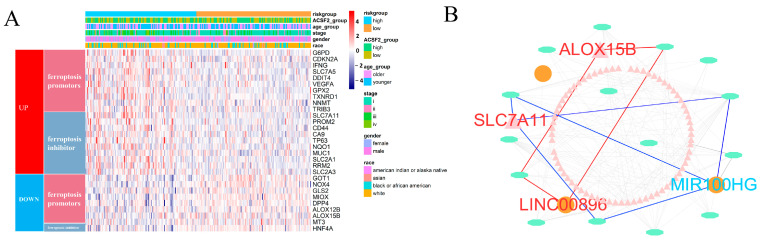
Comparison of ferroptosis activity and construction of ceRNA network related to FRM. (**A**) The differential expression of ferroptosis promoters and suppressors between KIRP samples with high and low FRM risk. (**B**) CeRNA network related to FRM. The triangles indicate mRNAs, ovals represent miRNAs, and squares represent lncRNAs. Among them, FRG (*ALOX15B* and *SCL711A*) and FRL (*LINC00896*), marked in red, were all upregulated. And, another FRL (*MIR100H*), marked in blue, was downregulated.

The expression pattern of ferroptosis regulators was compared between KIRP patients with high and low FRM risk scores. A total of 10 antiferroptosis regulators were upregulated in the FRM high-risk group, and 8 ferroptosis promoters were upregulated in the FRM low-risk group. (Figure 5A). Among these, *ALOX15B* was downregulated in the FRM high-risk group, while *SLC7A11* was upregulated (Figure 5B and Figure S8A). Next, we constructed a ceRNA network based on ferroptosis-related mRNA and nine lncRNAs involved in FRM (Figure S8B). Results showed that *SLC7A11* and *ALOX15B* were also involved in the ceRNA network and interacted with *LINC00896* and *MIR100HG*, respectively. Recent studies have revealed that high expression of *SLC7A11* promotes tumor growth, partly by suppressing ferroptosis. This finding suggests that *SLC7A11* could be a potential target for cancer therapy. We found that *SLC7A11* was expressed at higher levels in KIRP tumor tissues as well as in the high-FRI group. This suggests that overexpression of *SLC7A11* promotes tumor progression and is associated with a poor prognosis. As the ceRNA of *SLC7A11*, *LINC00896* is also overexpressed in tumors. This suggests that the overexpression of *LINC00896* prevents miRNA from inhibiting *SLC7A11* expression, thereby promoting tumor progression. Additionally, high expression of *LINC00896* is associated with a poor prognosis.

The FRI was calculated for each sample, and based on the results, the samples were divided into two groups: FRI-high and FRI-low (Figure S8C). Notably, the risk of FRM was significantly increased in the group with high FRI scores (*p* < 0.001) (Figure S8D). This suggests that intrinsic resistance to ferroptosis may contribute to the unfavorable outcome of patients with high FRM risk scores.

### 2.6. Identification of the FRM Risk-Associated Mutation Landscape in KIRP Samples

**Figure 6:**
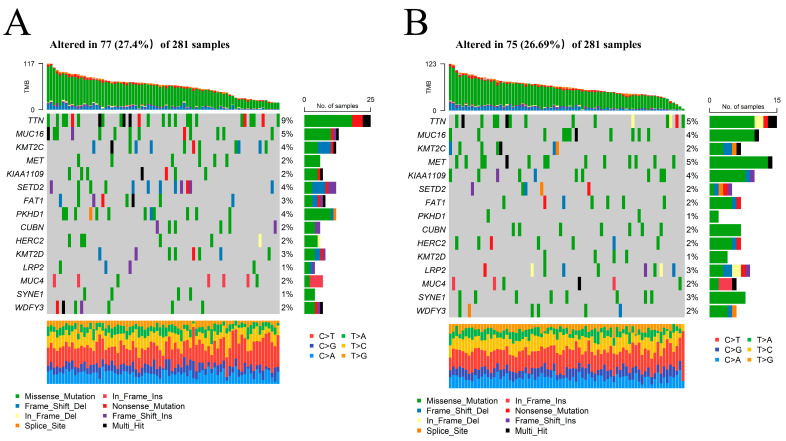
The mutation landscape between KIRP samples with high and low FRM risk. (**A**,**B**) Comparison of the mutation landscape between groups with high and low FRLM risk scores.

The mutation landscapes of the high- and low-risk groups in FRM were compared. The results showed that more mutation events occurred in samples with a higher risk of FRM. The predominant alteration in these samples was the mutation of TTN and MET (Figure 6A,B and Figure S9). Notably, the mutation rate of those two genes for KIRP was inconsistent between the two groups. MET, instead of TTN, had a higher mutation rate in samples with lower FRM risk scores. This finding may explain the relative survival benefits observed in KIRP patients. Studies have shown that MET mutations are an important mechanism in the pathogenesis of some PRPP patients. Therefore, FRM typing is beneficial for guiding medication for PRPP patients.

We also compared the mutations of ferroptosis genes and found that the mutation frequency was not high. The top three mutated ferroptosis genes were *NF2*, *BAP1*, and *ACSF2* (Figure S10). Correlation analysis of the expression of mutated ferroptosis genes revealed a strong correlation between *ACSF2* and the expression of several genes (Figure S11). Therefore, we concluded that *ACSF2* is an important prognostic factor for KIRP.

### 2.7. Comparison of the Sensitivity to Anticancer Drugs between Patients with Different FRM Risk Scores

**Figure 7:**
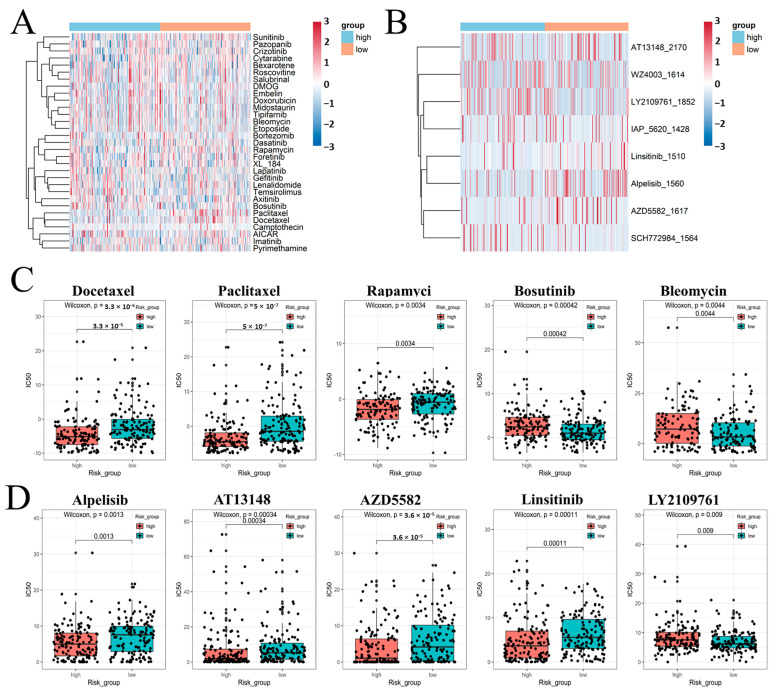
(**A**,**B**) Estimated drug sensitivity in patients with high and low FRM risk was calculated using R package ‘pRRophetic’ and ‘oncoPredict’. (**C**,**D**) Use box graphs to show the results of significant differences.

Next, we analyzed different drugs that have different potentials to be applied in the treatment of two groups of FRM-risk KIRP cancer in the future using two different R packages (‘pRRophetic’, version: 0.5, and ‘oncoPredict’, version: 0.2). The sensitivity to 30 common anticancer drugs and some candidate drugs was compared between the high- and low-risk groups to determine potential treatment modalities for renal cancer (Figure 7A,B). The results demonstrated that the IC50s of docetaxel, paclitaxel, rapamycin, alpelisib, AT13148, and AZD5582 were lower in patients with a higher FRM risk. This suggests that there is a higher sensitivity to these drugs following an increased FRM risk (Figure 7C,D). The IC50s of bosutinib, bleomycin, and Ly2109761 were higher in patients with a higher FRM risk.

### 2.8. Singer-Cell Analysis

**Figure 8:**
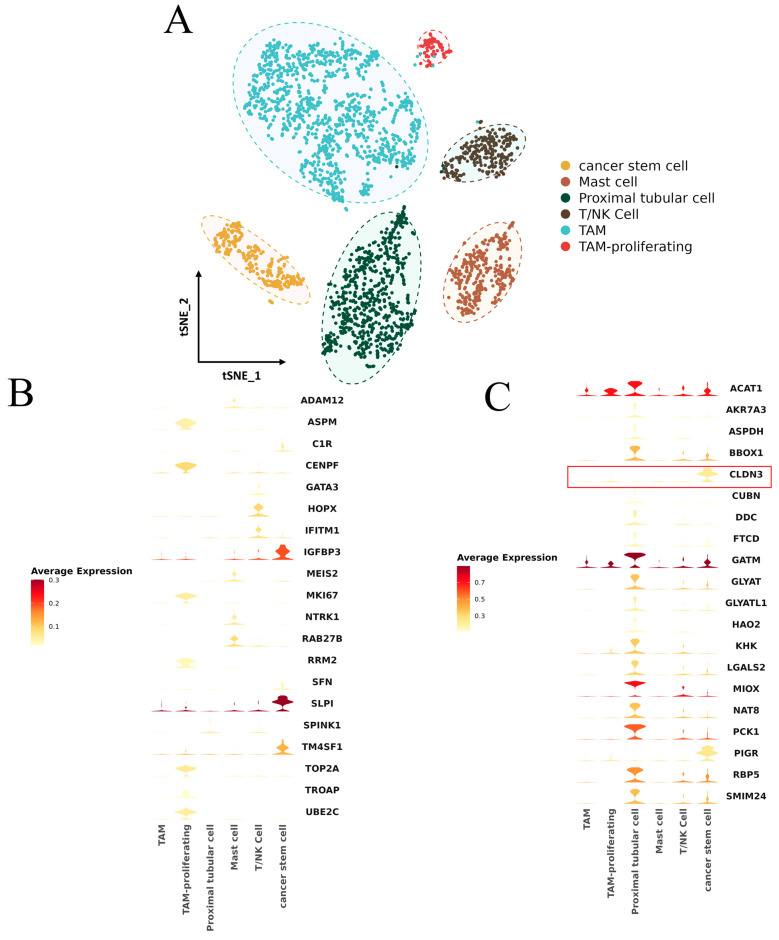
(**A**) KIRP single-cell subpopulation annotation t-SNE diagram. (**B**) Upregulation of gene expression profile in the high-FRM-risk group. (**C**) Upregulation of gene expression profile in the low-FRM-risk group, CLDN3 was marked with a red box.

To investigate the tumor development mechanism of KIRP and the relationship between FRM and tumor immune infiltration, we conducted an analysis of KIRP single-cell sequencing data using the R package “Seurat” (version: 3.4.0.1). The analysis involved dimensionality reduction clustering (dim = 1:13, resolution = 0.4), and we annotated six cell populations, namely tumor stem cells, renal tubular cells, mast cells, tumor-associated macrophages, proliferating tumor-associated macrophages, and T/NK cells (Figure 8A).

Cell-level expression analysis was conducted on high-risk differentially expressed genes of FRM and genes with a strong lncRNA correlation (Figure 8B,C). The results showed that the genes with high expression in the low-FRM-risk group were primarily found in renal tubular cells and tumor stem cells. On the other hand, the genes with high expression in the high-FRM-risk group were mainly observed in tumor stem cells and proliferating tumor macrophages. 

We also noticed that the gene encoding the tight junction protein 3 (Claudin3/CLDN3), a potential risk factor for tumor cell infiltration and metastasis [42], is specifically expressed in tumor stem cells and the expression level of this gene is higher in the low-risk group compared to the high-risk group (Figure 8B,C).

## 3. Discussion

Ferroptosis may be a promising solution for the treatment of multiple cancers in the future [43,44]. As ferroptosis is a totally different cell death process from apoptosis, ferroptosis reagents may represent a promising strategy for overcoming the inefficiency of apoptosis-inducing chemotherapy drugs in cell death induction [45]. In some cases, this metabolic reprogramming has been linked to an acquired sensitivity to ferroptosis, thus opening up new opportunities to treat therapy-insensitive tumors [46]. KIRP is the most heterogeneous form of kidney cancer, characterized by being hard to detect, having high malignancy, poor prognosis, and being difficult to treat [47]. The treatment of patients with advanced or relapsing KIRP faces significant challenges. The pathogenesis of KIRP is not fully understood, which poses a significant challenge to its treatment. Currently, the clinical treatment of KIRP typically involves surgical treatment, chemotherapy, and immunotherapy. However, the effectiveness of these methods is unsatisfactory. Therefore, we urgently need new biomarkers for the early diagnosis of KIRP and a reliable framework to guide the treatment of individual patients. In this context, ferroptosis-targeted treatment might be beneficial to KIRP. However, the regulatory mechanism of ferroptosis in KIRP remains largely unclear, especially in the area of non-coding RNAs, particularly lncRNAs. Various non-coding RNAs involved in the regulation of ferroptosis have been identified [48,49]. For example, the lncRNA *P53RRA*, which is downregulated in cancers, interacts with Ras GTPase-activating protein-binding protein 1 (G3BP1) and displaces p53 from the G3BP1 complex, causing p53 accumulation in the nucleus and ultimately resulting in ferroptosis and apoptosis [50]. *LINC00336* was reported to inhibit ferroptosis in lung cancer cells by acting as a sponge for *miR-6852* and positively regulating its target, CBS (cystathionine β-synthase) [51]. However, the regulatory network between ferroptosis regulators and lncRNAs in KIRP is likely to be extensive and complex. Therefore, systematic screening for potential FRLs is essential for accelerating KIRP treatment.

In this study, a matrix of ferroptosis regulators and lncRNAs was constructed and analyzed. The *ACSF2*-*LINC01020* pair was found to have a strong correlation in kidney cancer, suggesting that *ACSF2* may potentially promote ferroptosis in KIRP. Furthermore, *ACSF2* was found to be significantly downregulated in most tumors, particularly in renal cancer. We hypothesize that *ACSF2* may protect the body from tumors by acting as a cancer suppressor, which needs further research. Ferroptosis pathways are universally reprogrammed in multiple cancers, and several recent studies have constructed clinical prognostic models to predict the survival outcomes in cancer patients using the transcriptomic expression levels of ferroptosis regulators [24,52,53,54]. Zixuan Wu et al. [25] constructed a prognostic model of ferroptosis-related lncRNAs in KIRP using lasso regression. However, all patients, regardless of their risk level, had the same risk score, and the AUC of the signature lncRNAs was 1. This suggests that the model may not be appropriate. Herein, we generated a new ferroptosis regulator-lncRNA model using 9 lncRNAs and demonstrated its promising diagnostic accuracy for potential clinical translation (area under the curve = 0.91 for the 3-year survival rate). The molecular subtypes classified by the risk scores of the model did not belong to any previously reported subtypes of KIRP and exhibited the highest diagnostic accuracy.

Recent studies have revealed that the tumor microenvironment, particularly its immune cells, dictates whether tumor-cell ferroptosis will occur. In activated Treg cells, GPX4 could be induced and then resisted ferroptosis, which leads to tumor immune escape [55]. Cancer-associated fibroblasts (CAFs) exosome-derived *miR-3173-5p* sponged ACSL4 and suppressed ferroptosis, resulting in gemcitabine (GEM) resistance in pancreatic ductal adenocarcinoma (PDAC) [56]. Furthermore, immune checkpoint inhibitors (ICIs) and cyst(e)inase together potentiated T cell-mediated antitumor immune responses by synergistically promoting tumor ferroptosis [57]. In this study, we also discovered a correlation between the risk score calculated by FRM and metabolic rewiring, as well as the immune microenvironment. This correlation may provide guidance for different treatment approaches in the two groups. For example, more cancer-associated fibroblasts (CAFs) infiltrated the high-risk group of FRM, and the expression of *CD44* was also upregulated in this cluster. This upregulation may suppress ferroptosis in cancer cells, thereby increasing the risk of cancer. Therefore, immune checkpoint inhibitors that target *CD44*, such as SPL-108 [58] and hyaluronic acid [59], may be an optimal treatment modality for these patients.

Single-cell analysis suggests that the high-FRM-risk group had higher levels of immune cell infiltration. Both high-risk and low-risk groups have a certain number of tumor stem cells, and these cells possess the ability to self-renew and reproduce. This characteristic can contribute to the progression of cancer, which may explain the challenges encountered in KIRP treatment. The difference is that the expression level of tumor stem cell-related genes is higher in the low-FRM-risk group. This indicates that even if patients are at low FRM risk, their tumor development is in a rapid stage and there is still a trend of deterioration. Furthermore, we found that the gene encoding the tight junction protein 3 (Claudin3/CLDN3) is specifically expressed in tumor stem cells. The main function of CLDN3 is to maintain the physical barrier function and cell polarity between cells. If expressed abnormally, it can lead to the loss of cell adhesion and is a potential risk factor for tumor cell infiltration and metastasis [60]. This discovery opens up the possibility of using CLDN3 as a targeted therapy for malignant tumors [61]. The expression level of this gene is higher in the low-risk group compared to the high-risk group. This suggests that the low-risk group, in the early stages of tumor development, is more suitable for targeted treatment of CLDN3. However, the expression level of this gene is lower in the high-risk group, suggesting that tumor cells may have metastasized. As a result, targeted treatment of CLDN3 may no longer be appropriate.

The present study has several strengths. First, this study systematically investigated the correlation between ferroptosis genes and lncRNAs in KIRP. We also utilized GEO data and conducted pan-cancer analysis to validate the ferroptosis gene *ACSF2* as a novel negative regulator of ferroptosis induction. Second, we developed a ferroptosis-related lncRNA risk score model (FRM), which demonstrates high diagnostic accuracy and is valuable for clinical translation. Based on this model, we constructed a ferroptosis-related ceRNA network, indicating that *MIR100HG* might be a novel positive regulator of ferroptosis induction by inhibiting *SLC7A11*. Third, we employed two methods to compare the sensitivity of patients to 30 commonly used anticancer drugs and 8 potential anticancer drugs in high- and low-risk populations. These methods provide guidance to clinicians in selecting appropriate drugs for the treatment of KIRP. However, the current algorithm is based on cell lines and requires further validation through preclinical studies. Finally, we utilized KIRP single-cell sequencing data to compare the expression levels of various genes in high-risk and low-risk groups across five cell types. We found that the most highly expressed genes in the high-risk population were on tumor-associated macrophages, suggesting a close relationship between our FRM typing and tumor immunity. Leukemia is also a highly heterogeneous form of cancer, and the prognosis of some types remains poor. Compared to other cancer cells, renal cancer cells and leukemia cells are more susceptible to clearance. Some studies on leukemia have shown that targeted therapy has a positive effect on the treatment of leukemia [62,63], so our future research may be extended in the direction of leukemia treatment. However, it is important to note that the present study has limitations, primarily related to the use of transcriptome sequencing data and the relatively small sample size of single-cell data. In addition, a more efficient method for screening FRG is to directly compare and analyze samples that are sensitive to ferroptosis, rather than relying on correlation. Currently, no clinical drugs have been approved for inducing ferroptosis. However, we believe that the targeted drugs specifically designed to induce ferroptosis will be clinically approved in the future. At that time, we will be able to verify the feasibility of this screening strategy.

## 4. Materials and Methods

### 4.1. Collection of Data

The RNA-seq transcriptome data, which consisted of 289 KIRP samples and 32 adjacent normal tissues, as well as the corresponding clinical data, were obtained from the Cancer Genome Atlas data portal (TCGA, http://cancergenome.nih.gov/, accessed on 11 March 2023). The mutation data of the included KIRC samples were downloaded from TCGA in the maf format and analyzed subsequently using the R package ‘maftools’ (version 2.6.0). Clinical information of KIRP patients, including age, sex, grade, OS, survival status, and metastasis, was also extracted for subsequent analysis. The renal cell carcinoma RNAseq data from the Gene Expression Omnibus (GEO) database (GSE6344_GPL96, GSE25674, and GSE15641) were used as a validation cohort to identify differentially expressed FRGs. Among the datasets analyzed, DGSE6344_GPL96 included 10 tumor samples and 10 normal samples, GSE25674 included 8 tumor samples and 8 normal samples, and GSE15641 included 32 tumor samples and 23 normal samples. The data for single-cell RNA sequencing analysis was downloaded from PMC6104812. The fraction related to KIRP was extracted for analysis.

### 4.2. Identification of FRLs and DEFRGs

The FRG was extracted from FerrDb [64] and some references [44,65,66,67]. We extracted a total of 241 FRG from humans. Among these, 228 FRGs were obtained from FerrDb, including 71 drivers, 96 markers, and 61 suppressors. Additionally, 69 FRGs were obtained from four other references (Table S1), although some of them may be repetitive. Pearson’s correlation analysis was performed between FRGs and all lncRNAs. The lncRNAs with FRGs correlation coefficients greater than 0.3 and *p* values less than 0.001 were considered FRLs. Among these FRLs, those with correlation coefficients greater than 0.9 were sorted out along with their corresponding FRGs. To identify the DElncRNA, we used the R package ‘limma’ (version: 3.54.2) for differential expression analysis among FRLs. The thresholds were set as a log fold change (FC) greater than 2, along with a false discovery rate (FDR) less than 0.01. The DEmRNAs (including FRGs) were also identified using the same method. We utilized the R package limma for conducting differential expression analysis on three GEO RNAseq datasets. The thresholds were established as a log fold change (FC) greater than 1, in addition to a false discovery rate (FDR) lower than 0.05. Robust rank aggregation was used for gene list integration to obtain the mRNA differential expression in all three groups using the R package ‘RobustRankAggreg’ (version 1.1).

### 4.3. Establishment of a Risk Model for Evaluating the Risk Score

Univariate Cox regression was performed to screen OS-related FRLs (*p* < 0.01), followed by lasso regression analysis. Lasso regression removes genes that have a risk of overfitting, as determined by the partial likelihood deviance and lambda value. The lambda value is determined by the smallest likelihood deviance, and the coefficient-lambda curve shows genes that are eligible when the lambda value is determined. Finally, 19 FRLs were selected for multivariate Cox regression, and OS was analyzed. The R packages ‘survival’ (version: 3.3-1), ‘survminer’ (version 0.4.9), and ‘glmnet’ (version: 4.1.3) were used in this section. The area under the curve (AUC) value of the prognostic models was also calculated and plotted as a curve. The calculation procedure was terminated when the curve reached the highest point (i.e., the maximum AUC value), and the model was regarded as the optimal candidate (R package ‘survivalROC, version 1.0.3’). The 1-year, 5-year, and 10-year receiver operating characteristic (ROC) curves of the model are depicted. To validate the cut-off point, we conducted a Kaplan–Meier analysis to demonstrate the difference in survival between patients in the high-risk and low-risk groups. *p* < 0.05 indicates statistical significance. The specific risk score values of each sample in the model were also visualized using the R package ‘survminer’ (version 0.4.9). We also defined the ferroptosis resistance index (FRI) by integrating 128 FRGs that inhibit the execution of ferroptosis using the algorithm Single Sample Gene-Set Enrichment Analysis (R package: GSVA, version: 1.46.0).

### 4.4. Functional Enrichment Analysis

R packages ‘clusterProfiler’ (version: 4.9.2) were used to perform the Gene Ontology (GO) enrichment analysis, including biological process (BP), the cellular component (CC), molecular function (MF), and Kyoto Encyclopedia of Genes and Genomes (KEGG) pathway analysis. ‘Pathview’ (version: 3.18.0) and ‘enrichplot’ packages (version: 1.18.4) were used to visualize the enrichment results. The cutoff criteria with a *p* value < 0.05 were considered statistically significant. Gene set variation analysis (GSVA) enrichment analysis was performed to estimate the differences in KEGG pathways between any two clusters. This analysis was conducted using the R package “GSVA”, which employs a nonparametric and unsupervised method to detect changes in pathway activation. [68]. The significant biological processes met the standard of an adjusted *p* value < 0.05.

### 4.5. Construction of the ceRNA Network

It is important to match the differentially expressed mRNAs, miRNAs, and lncRNAs according to the competing endogenous RNA (ceRNA) hypothesis [69]. The interactions between miRNAs and mRNAs were evaluated using miRTarBase (Release 7.0). The interactions documented in this database are supported by robust experimental evidence, such as reporter assay or Western blot. Furthermore, the candidate lncRNA-miRNA interactions were selected based on highly conserved microRNA family data in the miRcode database (miRcode 11). The interactions between FRLs (ferroptosis-related long non-coding RNAs) and miRNAs associated with ferroptosis were evaluated to construct the ceRNA (competing endogenous RNA) network. Cytoscape v3.8 software was used to visualize this network.

### 4.6. Estimation of Intratumoral Immune Cell Infiltration

To analyze the association between the FRM risk score and immune cell infiltration, we utilized well-established methods to calculate the level of immune infiltration in the included KIRP samples. These methods include EPIC, MCPcounter, QUANTISEQ_default, QUANTISEQ, CIBERSORT, ssGSVA, TIMER, and XCELL algorithms. The differences in immune infiltrating cell constitutions between high- and low-risk groups of the constructed FRM were analyzed using the Wilcoxon signed-rank test. The results are displayed in a box diagram. Spearman correlation analysis was performed to investigate the relationship between the risk scores and the infiltrated immune cells. The correlation coefficients of the results were visualized in a lollipop chart. This section used the R ‘packages’ ggplot2 (version: 3.4.2). The list of immune checkpoint genes was presented in the Supplementary Methods Section. The scores for myeloid-derived suppressive cells, cancer-associated fibroblasts, M2-polarized macrophages, and T-cell exclusion were calculated using the Tumor Immune Dysfunction and Exclusion database.

### 4.7. The Significance of FRM in Drug Sensitivity

To evaluate FRM in the clinic for KIRP treatment, the IC50 of commonly administered chemotherapeutic drugs in the TCGA project of the KIRP dataset were calculated using the R package ‘pRRophetic’ and ‘oncoPredict’. The algorithm allows users to predict the clinical chemotherapeutic response using only baseline tumor gene expression data. This is achieved by creating statistical models from the gene expression and drug sensitivity data obtained from cell lines in the Cancer Genome Project. The AJCC guidelines recommend 30 common antitumor drugs, such as Adriamycin, Vinblastine, Cisplatin, and Imatinib, for cancer treatment. The difference in the IC50s of common antitumor drugs between the high- and low-FRM-risk groups was compared using the Wilcoxon signed-rank test, and the results are presented as box plots.

### 4.8. Singer-Cell Analysis

Accurately localize the expression of tumor markers in KIRP tumors using single-cell analysis and investigate their mechanisms of action in the development and prognosis of KIRP. The dataset is from the literature PMC6104812, which contains single-cell sequencing results for KIRP. Due to the large amount of sequencing data, we used the Python package scan (version: 1.9.0) to integrate the data and obtain an h5ad file. Then, “Seurat” (version: 4.1.1) was used for single-cell transcriptome analysis, which includes quality control filtering, dimensionality reduction clustering, cluster cell type annotation, and target gene localization analysis. Finally, this study aims to explore the mechanism of the selected biomarkers in KIRP.

### 4.9. Statistical Analysis

Quantitative analyses were performed using appropriate statistical methods. Comparisons between two groups were evaluated using either the Student’s t-test or the Wilcoxon signed-rank test, depending on whether the samples met the requirement for parametric tests, whereas comparisons among more than two groups were performed using one-way analysis of variance. For survival analysis, a Kaplan–Meier curve was first drawn, and the log-rank test was conducted to determine whether the difference in the OS of the two groups was significant. Correlations between two groups of numeric variables were tested using Spearman correlation. *p* < 0.05 was considered significant.

## 5. Conclusions

In conclusion, the present study constructed an FRM that exhibited high diagnostic accuracy in predicting overall survival in patients with KIRP. There are significant differences in tumor immune invasion, drug sensitivity, and cell heterogeneity between high-risk and low-risk patients. Future studies are expected to investigate the underlying regulatory mechanisms of how lncRNA regulates ferroptosis and its impact on the therapeutic efficacy of ferroptosis inducers. We hope that the utility of the constructed FRLM will also be validated in future clinical studies. 

## Data Availability

The public TCGA dataset (https://cancergenome.nih.gov/, accessed on 11 March 2023) was used to gather gene expression data for KIRP samples and clinical annotations. Additional RNA-seq data and clinical survival information were obtained from GSE6344_GPL96, GSE25674, and GSE15641 (https://www.ncbi.nlm.nih.gov/geo/, accessed on 18 March 2023). Data for single-cell RNA sequencing analysis were downloaded from PMC6104812.

## Supplementary Materials

The following supporting information can be downloaded at: https://www.mdpi.com/article/10.3390/cimb46030123/s1. Figure S1: Analysis of ferroptosis-related lncRNA. (A) Heatmap of DEFRL. (B) Volcano plot of DEFRL. (C) Metabolic reprogramming is involved in 208 FRGs associated with DEFRLs; Figure S2: (A) The ceRNA Sankey diagram of seven core ferroptosis regulators: *ACSF2*, *ANGPTL7*, *DUOX2*, *IFNG*, *PCK2*, *SOCS1*, and *TP63*. (B) Differential expression of *ACSF2*, *CA9*, *LOX*, *DDIT4*, and *CAV1* in three GEO databases. (C) Correlation analysis of *ACSF2* and *LINC01020* expression in KIRP, KICH, and KIRC. (D) Differential expression of *ACSF2* in pan-cancer; Figure S3: (A) ROC Curve of FRM based on multivariate Cox regression analysis. (B) The survival score of FRM was tested in different stages of the KIRP tumor using the Kruskal–Wallis test. The *p* value was calculated. (C,D) Kaplan–Meier curves of either the entire sample or randomly selected samples all showed a significant difference in OS between high and low FRM risks. (E) Multivariate Cox regression analysis revealed that the FRM risk score was an independent risk factor for OS in patients with KIRP. (F) The expression of *MIR100HG*, *MNX1_AS1*, and *LINC01016* in different stages of KIRP tumors; Figure S4: OS curves of nine predictive genes in KIRP; Figure S5: (A,B) The 842 genes were differentially expressed between the FRM high- and low-risk groups. (C–E) Gene Ontology (GO) analysis revealed 842 differentially expressed genes; Figure S6: (A–C) Gene Ontology enrichment analysis was performed on a total of 726 differentially expressed genes between the high- and low-risk groups; Figure S7: Multiple metabolic pathways were differentially activated between the high- and low-risk groups; Figure S8: (A,B) The expression of the FRG *ALOX15B* and *SCL711A* between high and low FRM risk. (C) Patients were classified into two groups based on the median value of the ferroptosis resistance index. (D) Patients in the high-risk group exhibited an increased resistance index to ferroptosis; Figure S9: The mutational landscape of KIRP includes variant classification, variant type, SNV class, variants per sample, variant classification summary, and the top 10 mutated genes; Figure S10: The mutation landscape of 39 FRG; Figure S11: The expression correlation of 39 FRG; Table S1: ferroptosis-related genes.
