# Supplementary material for: Ferroptosis-Related lncRNA to Predict the Clinical Outcomes and Molecular Characteristics of Kidney Renal Papillary Cell Carcinoma
Source: Curr Issues Mol Biol. 2024 Feb 29;46(3):1886–903. doi: 10.3390/cimb46030123 (PMC10969180; doi:10.3390/cimb46030123)
Supplement: Supplementary file 1 [file cimb-46-00123-s001.zip › Supplementary Figure.pdf]

Supplementary Material

Ferroptosis-related lncRNA to predict the clinical outcome and molecular characteristics of kidney renal papillary cell carcinoma

Yubo Gong [1†], Chenchen Zhang[1†], Hao Li[1†], Xiaojie Yu[1],Yuejia Li[1], Zhiguo Liu[1*], Ruyi He[1,2*]

1 School of Life Science and Technology , Wuhan Polytechnic University, WuHan, China;
2 State Key Laboratory of Biocatalysis and Enzyme Engineering, School of Life Sciences, Hubei University, China;
* Correspondence: hexiaoyi@outlook.com; zhiguo_l@126.com
† These authors contributed equally to this work

1. Supplementary Figures

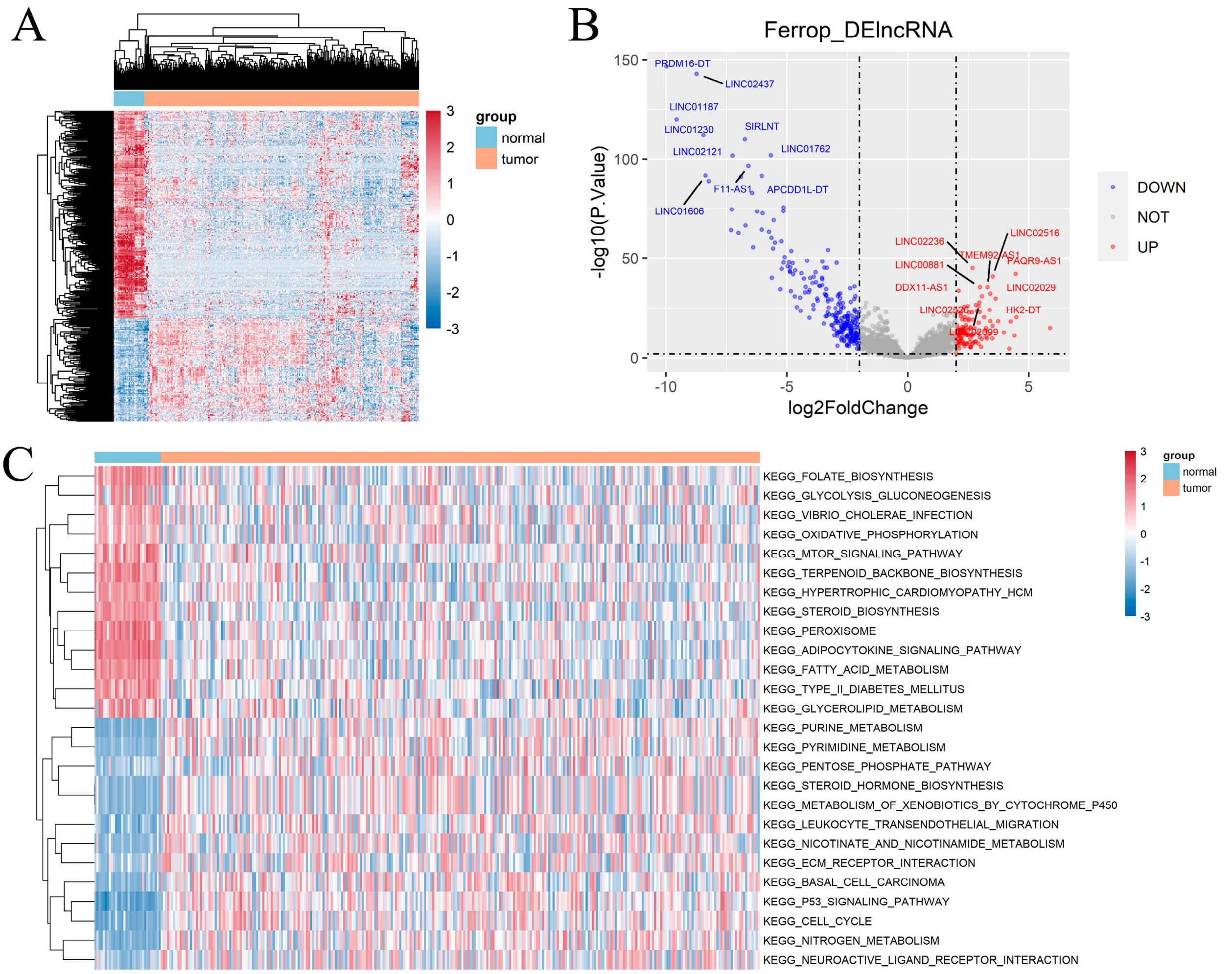

Figure S1. Analysis of ferroptosis-related lncRNA. (A) Heatmap of DEFRL. (B) Volcano plot of DEFRL. (C) Metabolic reprogramming is involved in 208 FRGs associated with DEFRLs.

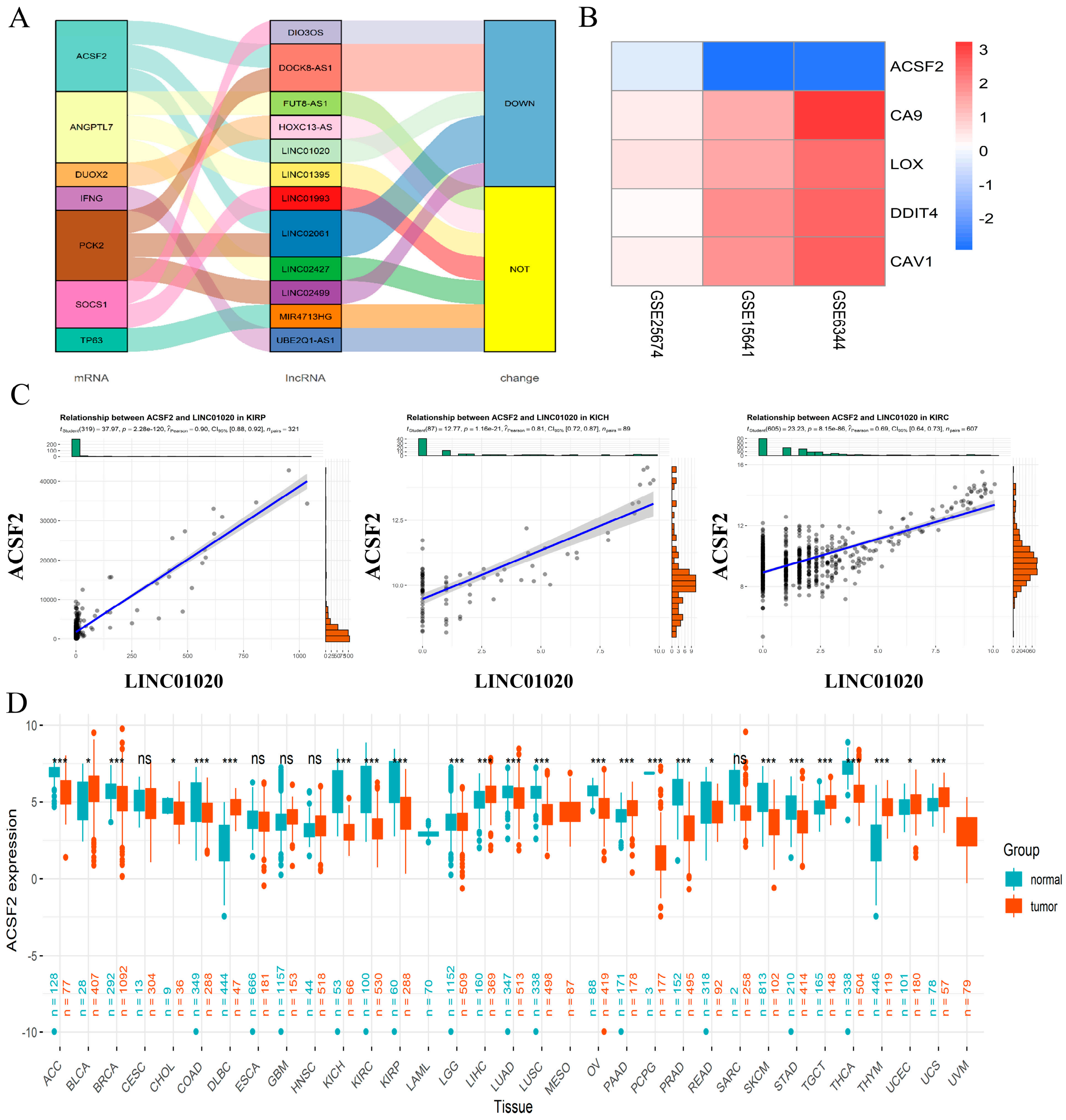

Figure S2. (A) The ceRNA Sankey diagram of seven core ferroptosis regulators: ACSF2, ANGPTL7, DUOX2, IFNG, PCK2, SOCS1, and TP63. (B) Differential expression of ACSF2, CA9, LOX, DDIT4, and CAV1 in three GEO databases. (C) Correlation analysis of ACSF2 and LINC01020 expression in KIRP, KICH, and KIRC. (D)Differential expression of ACSF2 in pan-cancer.

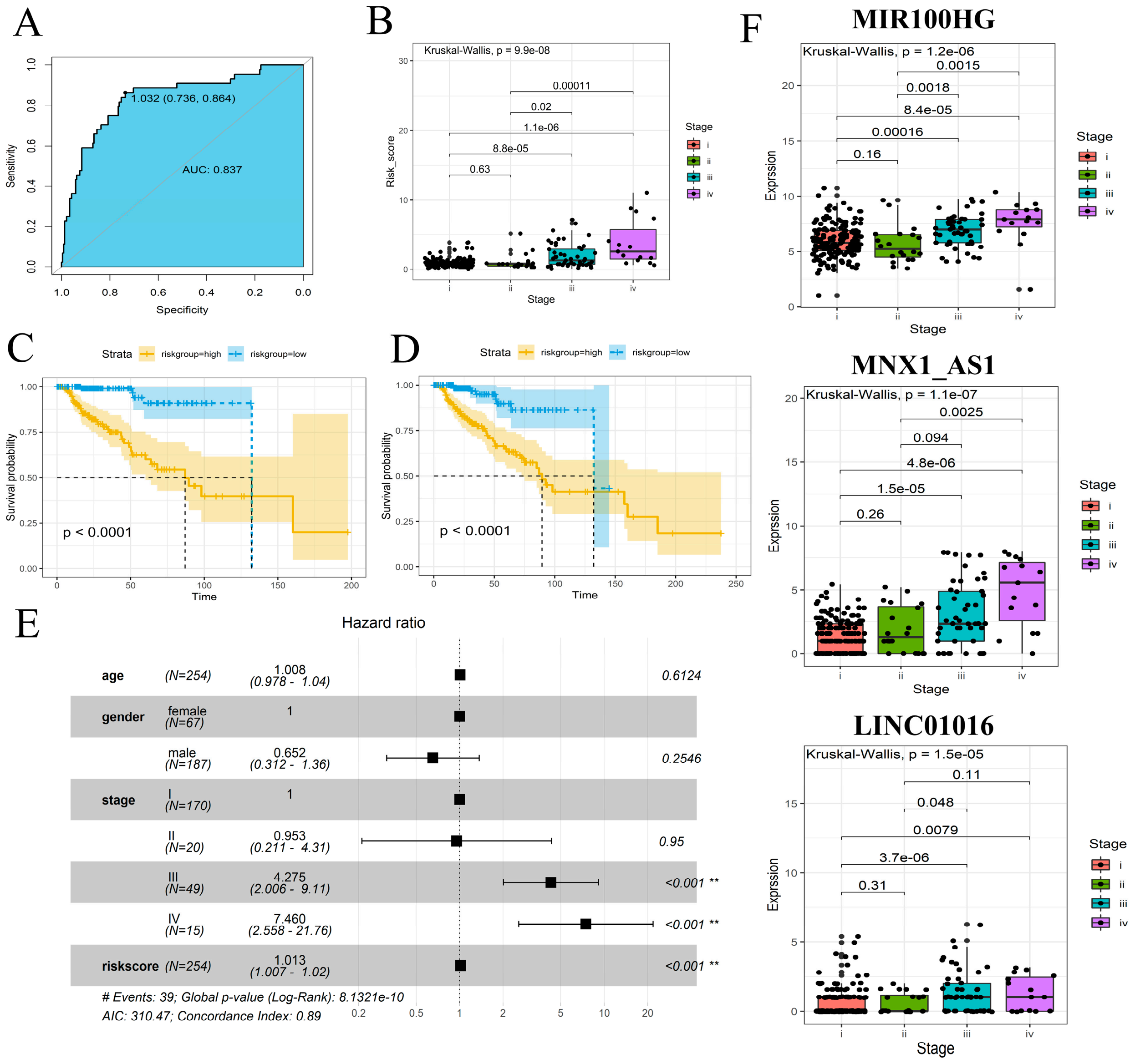

Figure S3. (A) ROC Curve of FRM based on multivariate Cox regression analysis. (B)The survival score of FRM was tested in different stages of the KIRP tumor using the Kruskal-Wallis test. The p-value was calculated. (C) and (D) Kaplan-Meier curves of either the entire sample or randomly selected samples all showed a significant difference in OS between high and low FRM risks. (E) Multivariate Cox regression analysis revealed that the FRM risk score was an independent risk factor for OS in patients with KIRP. (F) The expression of MIR100HG, MNX1_AS1, and LINC01016 in different stages of KIRP tumors.

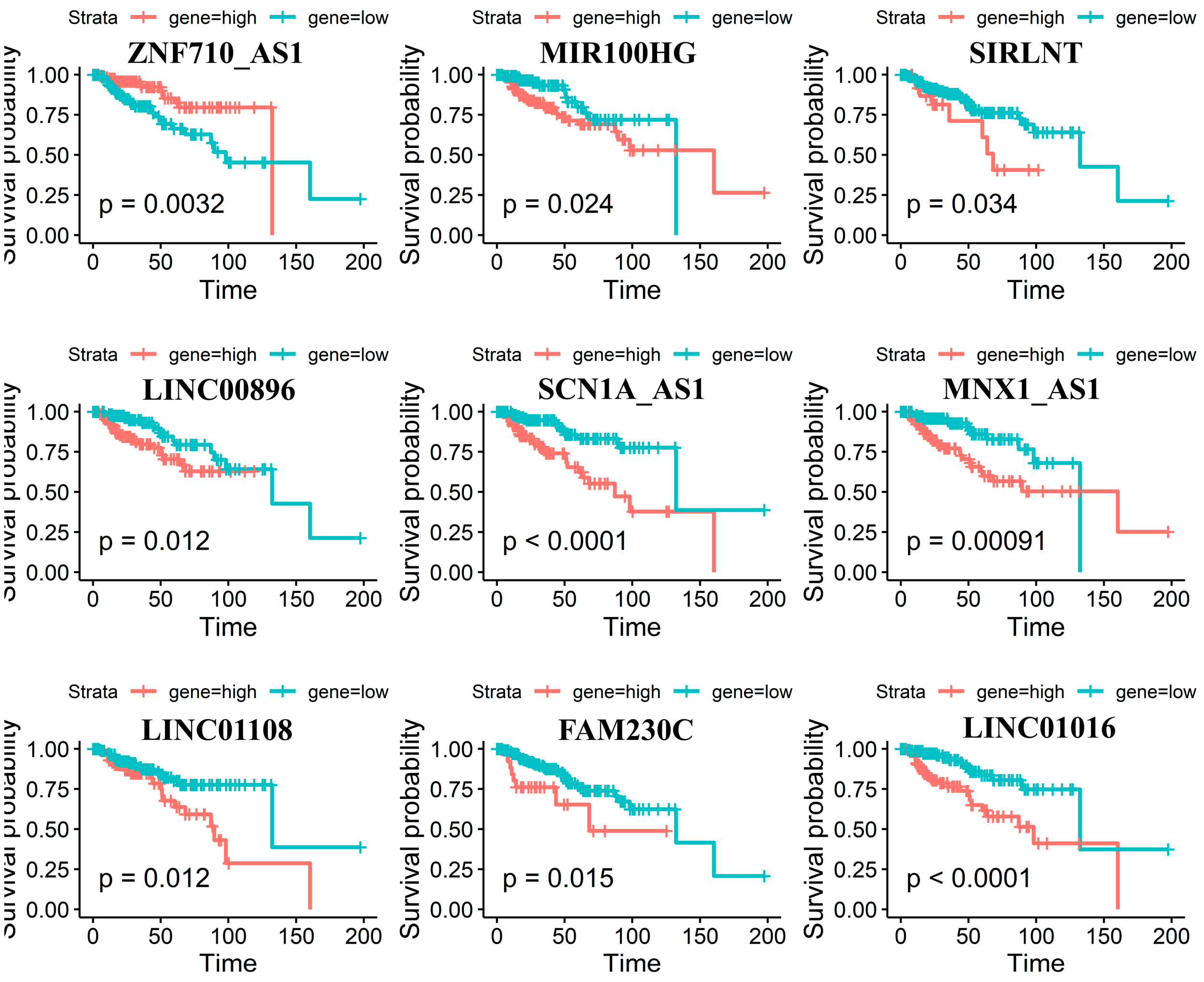

Figure S4. OS curves of nine predictive genes in KIRP.

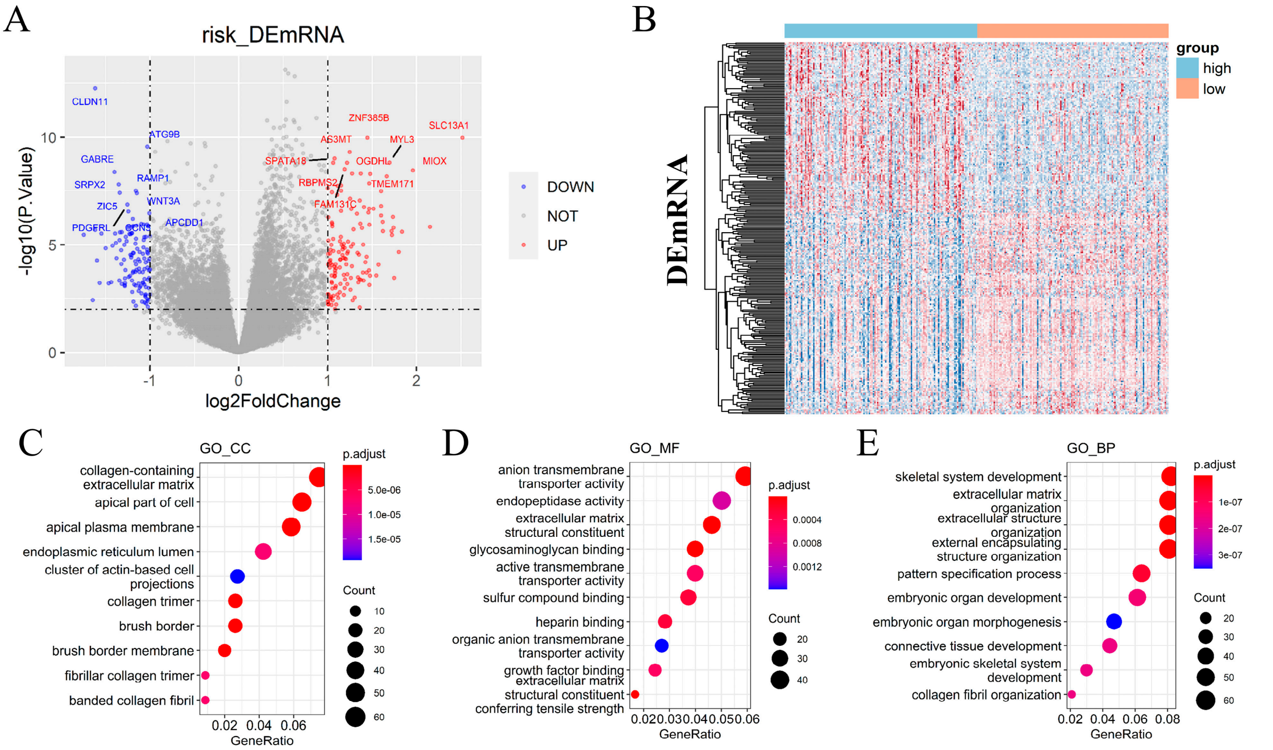

Figure S5. (A and B) The 842 genes were differentially expressed between the FRM high- and low-risk groups. (C to E) Gene ontology (GO) analysis revealed 842 differentially expressed genes.

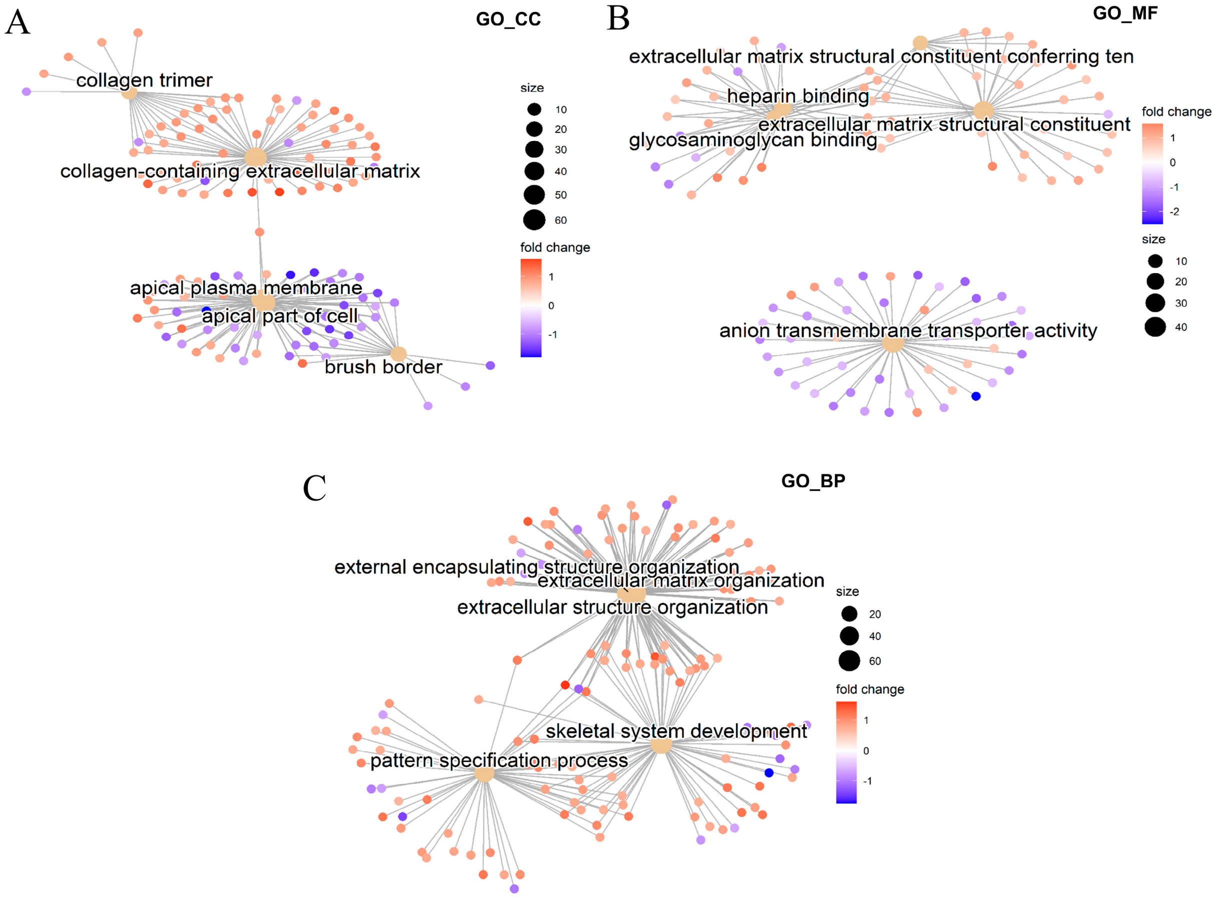

Figure S6. (A)-(C) Gene Ontology enrichment analysis was performed on a total of 726 differentially expressed genes between the high- and low-risk groups.

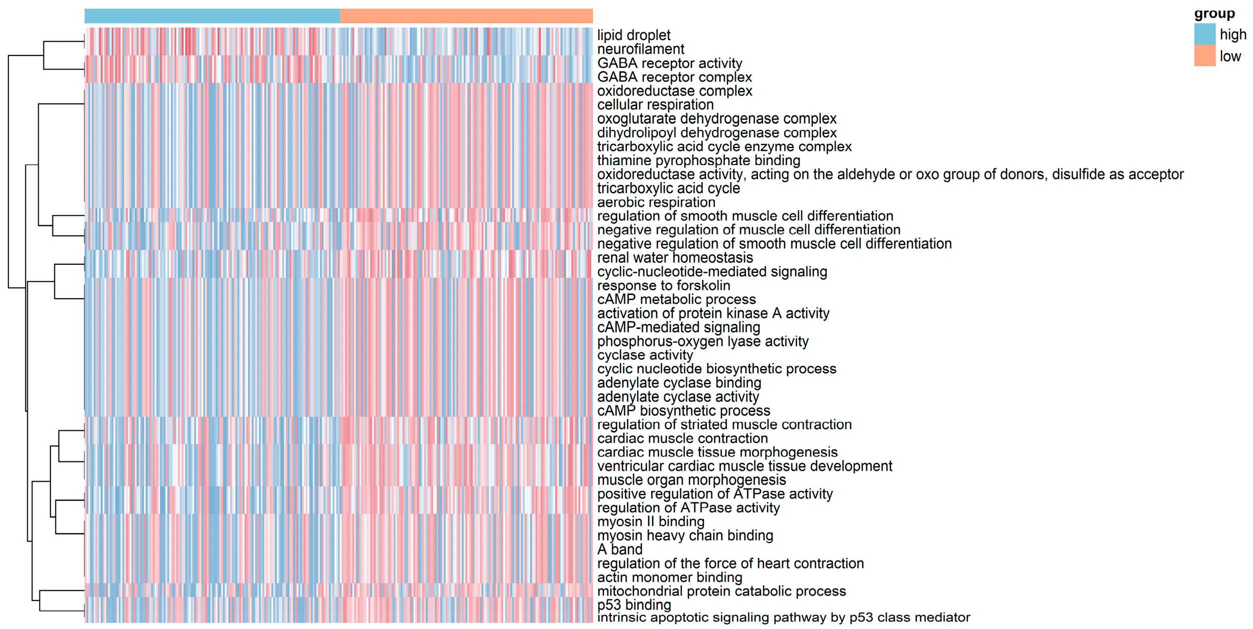

Figure S7. Multiple metabolic pathways were differentially activated between the high- and low-risk groups.

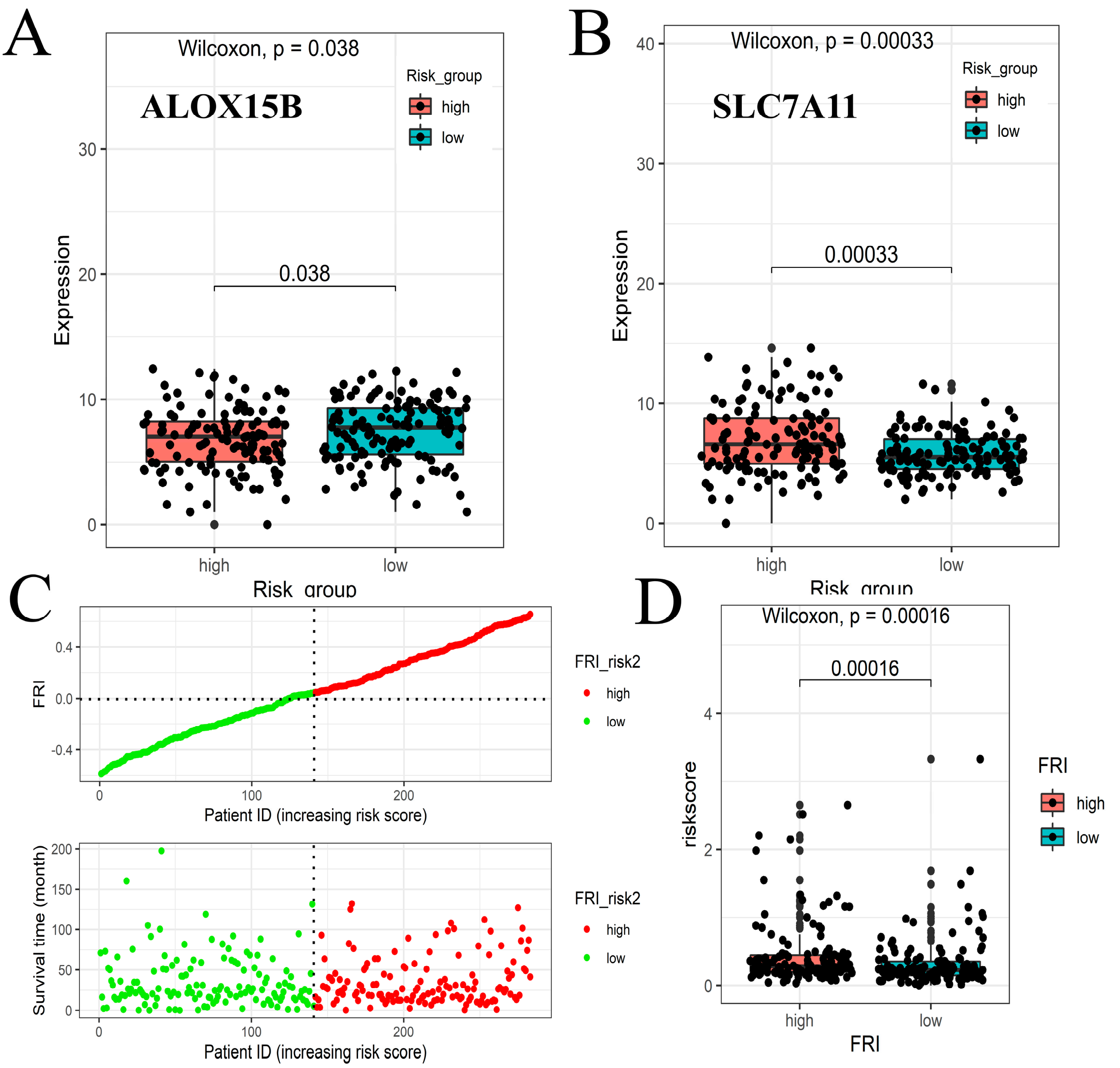

Figure S8. (A and B) The expression of the FRG ALOX15B and SCL711A between high and low FRM risk. (C) Patients were classified into two groups based on the median value of the ferroptosis resistance index. (D) Patients in the high-risk group exhibited an increased resistance index to ferroptosis.

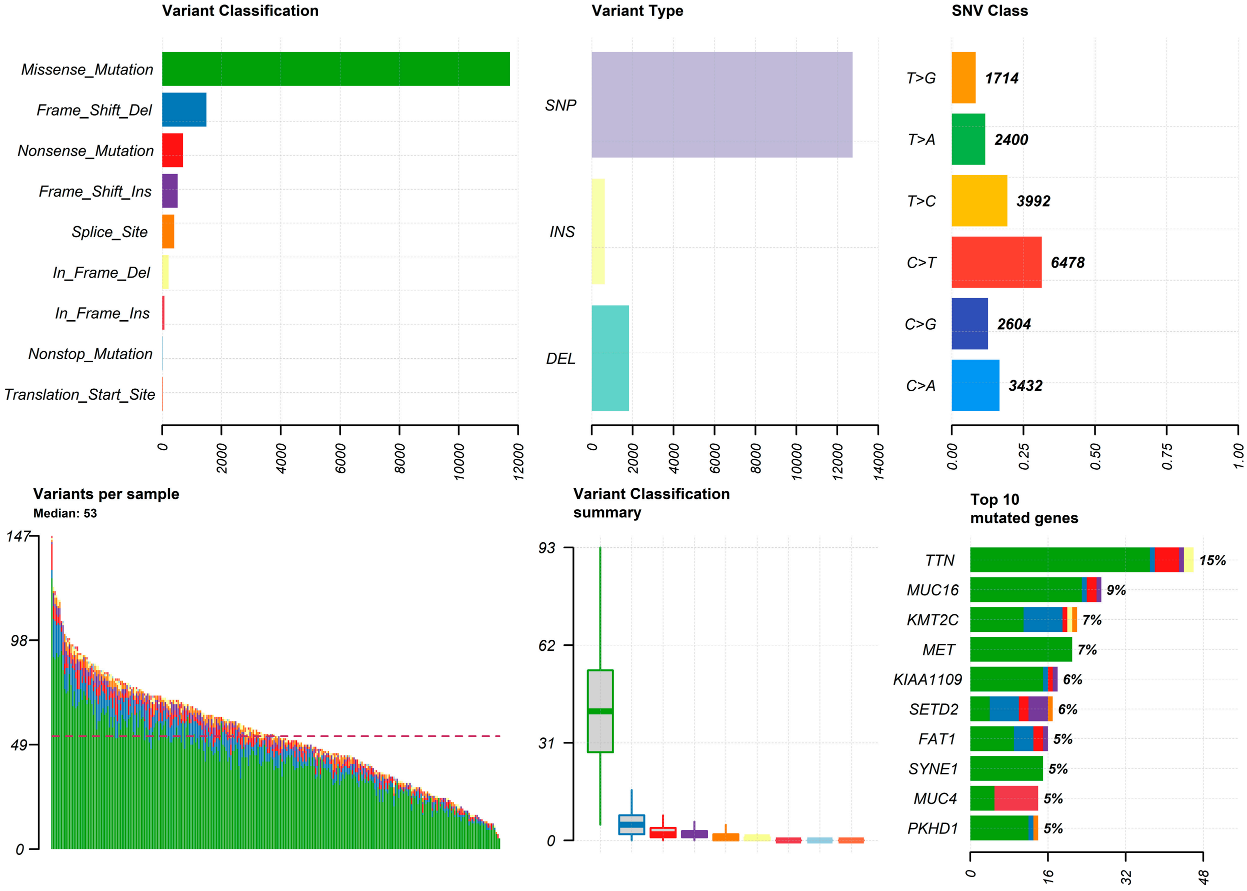

Figure S9. The mutational landscape of KIRP includes variant classification, variant type, SNV class, variants per sample, variant classification summary, and the top 10 mutated genes.

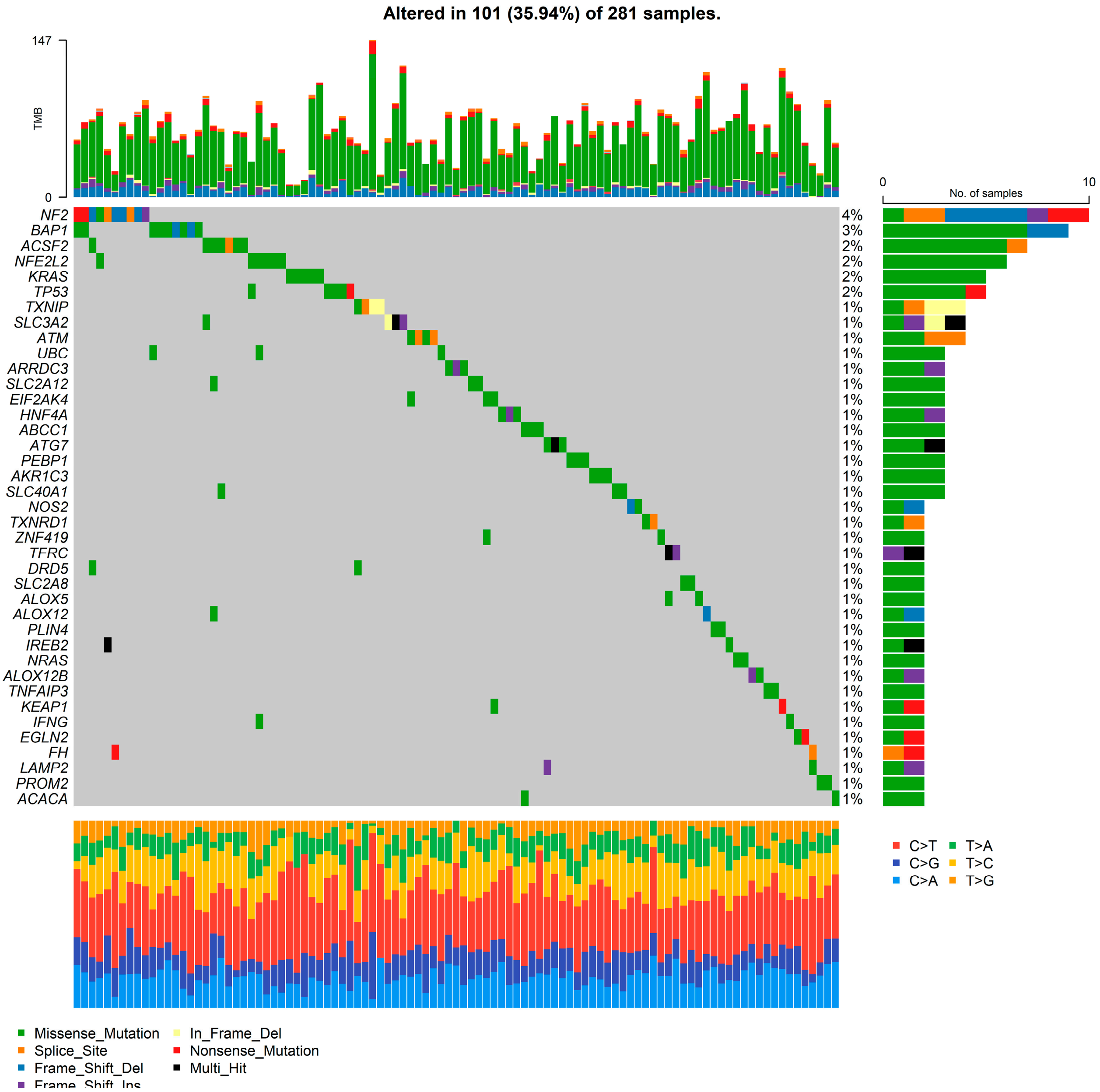

Figure S10. The mutation landscape of 39 FRG.

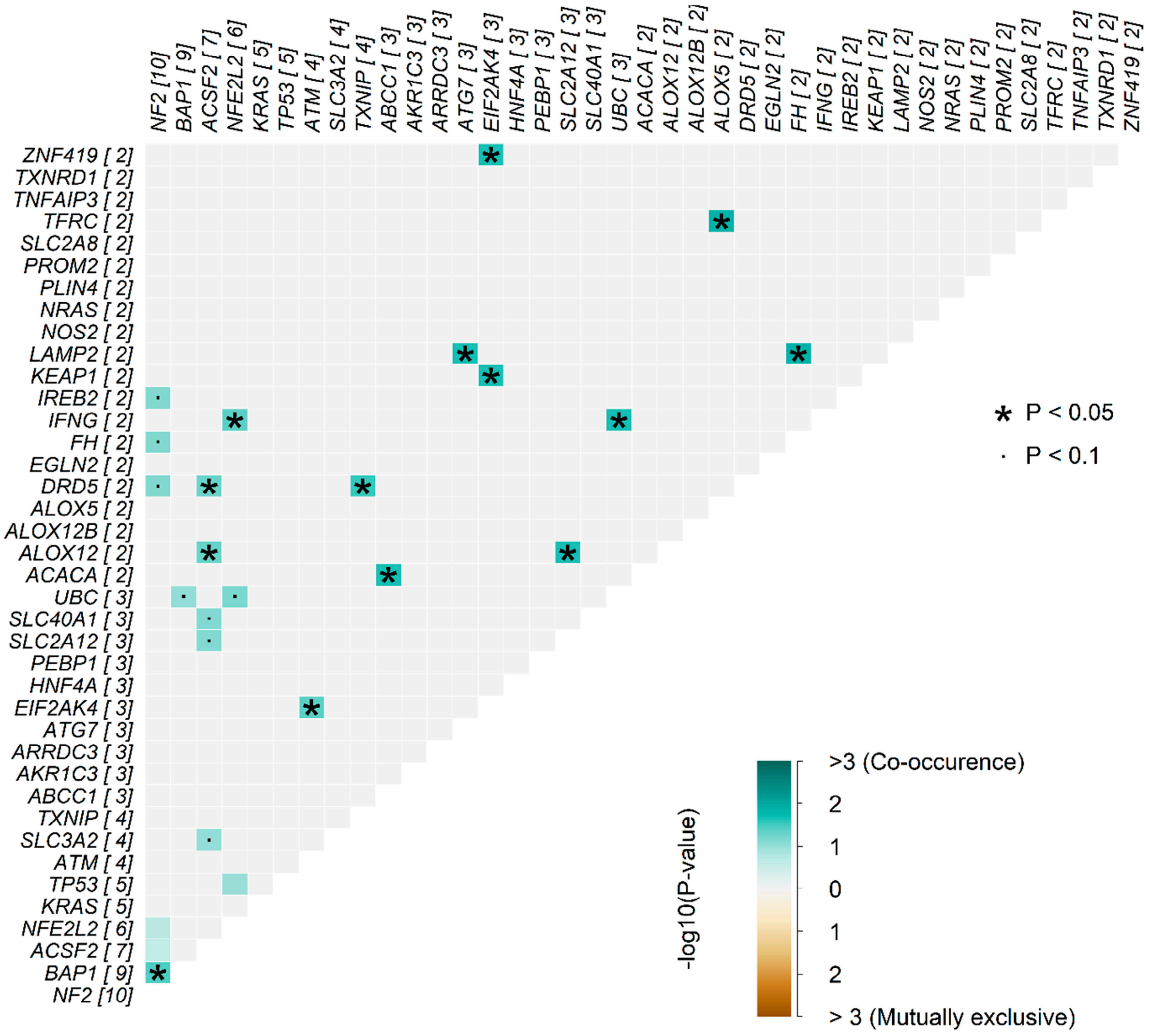

Figure S11. The expression correlation of 39 FRG.
